# Expression Silencing of Glutathione Peroxidase 4 in Mouse Erythroleukemia Cells Delays In Vitro Erythropoiesis

**DOI:** 10.3390/ijms22157795

**Published:** 2021-07-21

**Authors:** Marlena Rademacher, Hartmut Kuhn, Astrid Borchert

**Affiliations:** Charité—Universitätsmedizin Berlin, Corporate Member of Freie Universität Berlin and Humboldt-Universität zu Berlin, Institute of Biochemistry, Charitéplatz 1, 10117 Berlin, Germany; marlena.rademacher@charite.de (M.R.); hartmut.kuehn@charite.de (H.K.)

**Keywords:** cell differentiation, lipid peroxidation, oxidative stress, eicosanoids

## Abstract

Among the eight human glutathione peroxidase isoforms, glutathione peroxidase 4 (GPX4) is the only enzyme capable of reducing complex lipid peroxides to the corresponding alcohols. In mice, corruption of the *Gpx4* gene leads to embryonic lethality and more detailed expression silencing studies have implicated the enzyme in several physiological processes (e.g., embryonal cerebrogenesis, neuronal function, male fertility). Experiments with conditional knockout mice, in which expression of the *Gpx4* gene was silenced in erythroid precursors, indicated a role of Gpx4 in erythropoiesis. To test this hypothesis in a cellular in vitro model we transfected mouse erythroleukemia cells with a Gpx4 siRNA construct and followed the expression kinetics of erythropoietic gene products. Our data indicate that Gpx4 is expressed at high levels in mouse erythroleukemia cells and that expression silencing of the *Gpx4* gene delays in vitro erythropoiesis. However, heterozygous expression of a catalytically inactive Gpx4 mutant (*Gpx4*^+/Sec46Ala^) did not induce a defective erythropoietic phenotype in different in vivo and ex vivo models. These data suggest that Gpx4 plays a role in erythroid differentiation of mouse erythroleukemia cells but that heterozygous expression of a catalytically inactive Gpx4 is not sufficient to compromise in vivo and ex vivo erythropoiesis.

## 1. Introduction

Erythropoiesis is a multi-step process converting multipotent hematopoietic stem cells (HSC) to the mature peripheral erythrocytes circulating in the peripheral blood [1]. During the early steps of erythropoiesis HSC differentiate from the common myeloid progenitor cell of the megakaryocytic-erythroid linage to the burst-forming unit- erythroid (BFU-E). BFU-E cells are the first cellular progenitors committed solely to the erythroid lineage [2]. These BFU-Es further differentiate into the colony-forming unit erythroid (CFU-E) and this process concludes the first phase of erythroid differentiation. The second phase of erythroid differentiation involves the maturation of the nucleated precursors from the proerythroblasts to basophilic, polychromatic and orthochromatic erythroblasts. This phase is characterized by the gradual accumulation of hemoglobin, progressive decrease in cell size and nuclear condensation ultimately resulting in enucleation [1,2]. The final phase of erythropoiesis involves the development of the reticulocyte into mature erythrocytes. During this developmental stage erythrocytes acquire their characteristic biconcave shape and will be released into the bloodstream. Mature human red blood cells remain in circulation for about 120 days and during this time period residential macrophages in liver and spleen continuously survey them for structural and functional integrity [3]. Aged or damaged erythrocytes are phagocytosed by these macrophages and this concludes the life cycle of the red cells. In mammals terminal erythroid differentiation proceeds in erythroblastic islands within the bone marrow. These islands consist of a central macrophage, which is surrounded by up to 30 erythroid cells of variable degrees of maturation [4]. The central macrophage fulfills many functions [5,6,7]: (i) It anchors the erythroid cells within an erythroblastic island. (ii) It regulates the cellular interactions required for effective erythroid differentiation. (iii) It phagocytoses the nuclei extruded from erythroid precursors during terminal erythroid differentiation. (iv) It supplies the erythroid precursors with iron for effective heme synthesis. (v) It secretes cytokines and other signaling molecules regulating erythroid differentiation and cell proliferation in the immediate surrounding.

Glutathione peroxidases (Gpx-isoforms) form a family of enzymes capable of reducing organic and inorganic peroxides at the expense of reduced glutathione or alternative electron donors [8]. The human genome involves eight different functional GPX genes (*GPX1-8*), which encode for eight functionally distinct GPX-isoforms. In humans GPX1, GPX2, GPX3, GPX4 and GPX6 are selenoproteins carrying selenocysteine as catalytically active amino acid at the active site. The other human GPX-isoforms contain a cysteine at this position. The mouse genome involves an ortholog for each human gene but in this species the *Gpx6* gene encodes for a cysteine-containing enzyme. Among glutathione peroxidase isoforms GPX4 is somewhat unique since these enzyme orthologs are capable of reducing not only simple inorganic hydroperoxides such as H_2_O_2_ but also complex hydroperoxy ester lipids [9,10]. In humans and mice, GPX4 is expressed in three isoforms, which differ from each other with respect to their subcellular localization. cGPX4 is mainly localized in the cytosol, mGPX4 occurs in the mitochondria and nGPX4 prevails in the nucleus [11,12,13]. Although these isoforms are encoded for by the same gene (*Gpx4*) the primary translation products are protein-chemically distinct.

*Gpx4^−/−^* mice are not viable but die at early stages (E6.5) of embryogenesis [14,15] and this is also the case for mice expressing catalytically silent mutants (Sec46Ala, Sec46Ser) of the enzyme [16,17]. However, mice expressing the Sec46Ala mutant heterozygously are viable and do not show obvious phenotypic alterations [16]. More detailed studies of the reproduction behavior of these animals indicated that males are subfertile, which has been related to structural and functional defects of their sperm [18]. The molecular basis for the embryonic lethality of *Gpx4^−/−^* mice is still a matter of discussion but siRNA induced Gpx4 expression silencing during in vitro embryogenesis [19] and neuron specific disruption of the *Gpx4* gene [20] suggested a role of the enzyme in early cerebral embryogenesis. More recently, it was reported that conditional knockout of the *Gpx4* gene in hematopoietic cells compromised erythropoiesis of adult mice [21]. Functional inactivation of *Gpx4* gene induces necroptotic death of erythroid precursors, which leads to anemia that is partly compensated by increased extramedullary erythropoiesis [21]. If similar mechanisms proceed during early embryogenesis malfunction of the erythropoietic system may contribute to embryonic lethality of *Gpx4*^−/−^ mice.

To contribute to the discussion on the potential role of Gpx4 in erythropoiesis we employed mouse erythroleukemia cells (MEL), which differentiate along the erythroid linage [22,23] when stimulated with dimethylsulfoxide (DMSO). This cellular erythropoiesis model is frequently used and is considered a suitable cellular model for later stages of erythropoiesis [24,25]. Stimulated MEL cells reach the differentiation levels of nondividing normoblasts, synthesize hemoglobin but do not proceed into final erythroid differentiation [26,27,28]. For our studies, we first tested whether resting and DMSO-treated MEL cells express different Gpx4-isoforms. Next, we silenced Gpx4 expression in these cells using the RNAi technology (stable transfection with an anti-Gpx4 siRNA plasmid) and tested the impact of this genetic manipulation on DMSO-induced erythropoiesis. We found that Gpx4 isoforms are expressed in MEL cells and that expression silencing delays in vitro erythropoiesis. This data is consistent with the above referenced in vivo studies [21,29]. However, when we tested *Gpx4*^+/Sec46Ala^ mice, which heterozygously express a catalytically silent Gpx4 mutant, we did not observe a defective hematopoietic phenotype. Moreover, ex vivo erythropoiesis of embryonic liver cells did not reveal significant differences between wildtype and *Gpx4*^+/Sec46Ala^ hepatocytes. Taken together these data suggest that heterozygous expression of a catalytically silent Gpx4 mutant may not be sufficient to compromise in vivo erythropoiesis.

## 2. Results

### 2.1. Expression of Gpx4 Isoforms in Mouse Erythroleukemia Cells

**Figure 1:**
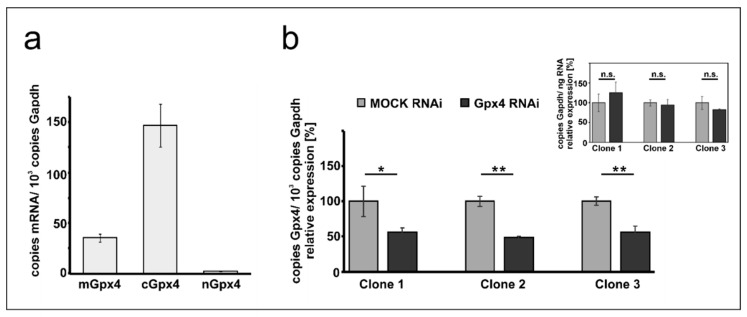
Expression of Gpx4 isoforms in native and transfected MEL cells. Total RNA was extracted from MEL cells, and the steady-state concentrations of mRNAs encoding for different Gpx4-isoforms were quantified by qRT-PCR using the specified amplification primers as described in the Material and Method section. (**a**) Expression of Gpx4-mRNA isoforms in native MEL cells. Each sample was quantified in triplicates. (**b**) Expression of Gpx4-isoforms in stably transfected RNAi MEL cells. Native MEL cells were transfected with a Gpx4-siRNA plasmid (Gpx4 RNAi) or with a corresponding control vector (MOCK RNAi). Different isolated growing cell clones were selected as described in Material and Methods. Expression of Gpx4-mRNA isoforms was quantified in three selected cell clones and Gpx4 mRNA copy numbers were related to the Gapdh mRNA copy numbers. Inset: Quantification of Gapdh mRNA in MOCK RNAi and Gpx4 RNAi MEL cells related to the amount of total RNA. RNA preparations of three different samples were analyzed and each sample was quantified in triplicates. For statistical comparison, the Student’s *t*-test was performed. * *p* < 0.05, ** *p* < 0.005; mGpx4: mitochondrial, cGpx4: cytosolic, nGpx4: nuclear isoform.

Mouse erythroleukemia (MEL) cells are frequently employed as cellular in vitro model for studies on mechanistic details of erythroid differentiation. Resting MEL cells do neither express hemoglobin nor other red cell specific proteins, but stimulation with DMSO induces erythroid differentiation reaching the level of orthochromatic normoblasts [28,30]. To explore whether these cells express Gpx4 isoforms we first carried out qRT-PCR to quantify the steady state concentrations of Gpx4 mRNA in resting MEL cells (Figure 1a). With more than 150 copies mRNA per 1000 copies of Gapdh mRNA, the cytosolic isoform (cGpx4) is expressed at high levels. The mGpx4 mRNA, which encodes for the mitochondrial Gpx4 isoform, was also present, but its expression level was 4-fold lower than that of cGpx4. The nuclear Gpx4 mRNA was only present in small amounts (less than 1% of cGpx4 mRNA). A similar Gpx4 expression pattern was previously observed in different mouse somatic tissues [16].

### 2.2. Stable Transfection of MEL Cells with Anti-Gpx4 Antisense Construct Reduced Gpx4 Expression

To explore the impact of expression silencing of Gpx4 we first established a cellular model, in which expression of the enzyme was compromised using the siRNA technology. For this purpose, we stably transfected resting MEL cells with an anti-Gpx4 anti-sense plasmid and analyzed a number of cell clones that survive the selection procedure. During the initial screen three well separated cell clones were selected and expression of the c + mGpx4 was quantified by qRT-PCR (Figure 1b). Here we found that in all selected cell clones Gpx4 expression was silenced by about 50%. To exclude that our transfection strategy impacted the expression of the reference gene we also quantified Gapdh expression in the three cell clones but did not observe significant differences when the Gapdh mRNA copy numbers were related to the amount of total cellular RNA (inset to Figure 1b). For detailed erythropoiesis studies we selected clone 2 since in these cells Gpx4 expression was most strongly (51 ± 2%) silenced.

### 2.3. Basic Characterization of Gpx4 Compromised MEL Cells

**Figure 2:**
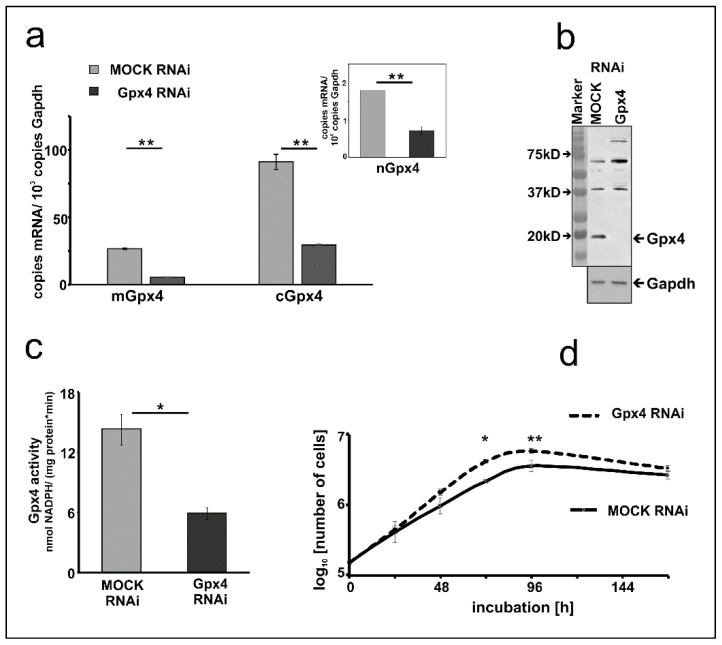
Expression silencing of Gpx4 in MEL cells stably transfected with an anti-Gpx4 siRNA. (**a**) Expression of Gpx4 mRNA isoforms in stably transfected RNAi MEL cells. Total RNA was extracted from MEL cells, and the steady-state concentration of mRNAs of the different Gpx4-isoforms was quantified by qRT-PCR using the specified amplification primers as described in the Material and Method section. Inset: Quantification of nGpx4 mRNA isoform. (**b**) Expression of Gpx4 protein in stably transfected RNAi MEL cells. MOCK RNAi and GPx4 RNAi MEL cells were homogenized, and aliquots (100 µg lysate supernatant protein) were applied to Western blotting using the monoclonal anti-human Gpx4 antibody (#MABF1969, Merck) [31] as described in Material and Methods. The absence of Gpx4 protein in GPx4 RNAi treated MEL cells was indicated by the absence of the immunoreactive band in the molecular weight range of about 20 kD. The chemical identity of the immunoreactive bands at higher molecular weight remains unclear. Since the monoclonal antibody employed for these analyses recognizes a specific amino acid sequence motif [31], which is also present in other mouse proteins. Thus, the most plausible explanation for the immunoreactive bands with higher molecular weights is cross-reactivity of the antibody with non-Gpx4 related proteins. Expression of the Gapdh protein was quantified as loading control. (**c**) Gpx4 activity in stably transfected RNAi MEL cells. Cells were homogenized, and the Gpx4 activity was assayed in the 10,000 g cell lysate supernatant using purified phosphatidylcholine hydroperoxide as substrate (see Material and Methods). (**d**) Cell division rate of stably transfected RNAi MEL cells. MEL cells, stably transfected with a Gpx4 siRNA plasmid (Gpx4 RNAi) or with a corresponding control vector (MOCK RNAi), were seeded in culture dishes (10^4^ cells/mL) in triplicates and cultured at 37 °C for 7 days as described in Material and Methods. Cells were counted daily. Significances were calculated using the Student’s *t*-test, n = 3, * *p* < 0.05, ** *p* < 0.005.

To characterize the Gpx4 deficient MEL cells in more detail we first quantified expression silencing of the two major Gpx4 isoforms on the mRNA level. Here we found that the Gpx4 expression levels (cGpx4 + mGpx4) in wildtype MEL cells (about 200 copies of m + cGpx4 mRNA per 1000 Gapdh mRNA copies, Figure 1a) were somewhat higher than those in the mock-transfected cells (about 120 copies of m + cGpx4 mRNA per 1000 Gapdh mRNA copies, Figure 2a). These data suggested that the Gpx4 expression level was somewhat reduced by the transfection procedure. Next, we quantified the different Gpx4 mRNA isoforms (cGpx4, mGpx4, nGpx4) in wild type MEL cells (Figure 1a), in MEL cells transfected with a Gpx4-siRNA plasmid (Gpx4 RNAi) or with a corresponding control vector (MOCK RNAi) (Figure 2a) and observed similar expression patterns. In all cell types cGpx4 was dominant followed by mGpx4. The nuclear isoform (nGpx4 mRNA) was always present in small amounts. Most importantly, when we compared the steady state concentrations of the Gpx4 isoforms in mock-transfected cells with those in cells transfected with the anti-Gpx4 antisense plasmid we found significantly reduced mRNA concentrations (>50% reduction) for all isoforms (Figure 2a). This was even the case for nGpx4 (Figure 2a, inset), which was only present in small amounts. 

To explore whether Gpx4 expression silencing could also be detected on the level of the Gpx4 protein we carried out immunoblotting. Cell lysis supernatants of mock-transfected MEL cells and corresponding lysates supernatants of MEL cells transfected with the anti-Gpx4 antisense constructs were analyzed by SDS-PAGE and the blots were probed with an anti-human GPX4 antibody, which cross-reacts with the mouse ortholog [31]. As shown in Figure 2b an immunoreactive protein with an apparent molecular weight of 18 kDa was detected in the lysis supernatant of mock-transfected cells. This band was not visible anymore when the lysate supernatant of MEL cells was analyzed, which were transfected with the anti-Gpx4 antisense plasmid.

Next, we carried out activity assays with the lysate supernatants of mock-transfected and antisense-transfected cells. For specific measurements of the Gpx4 activity we employed HPLC purified hydroperoxy phosphatidylcholine as substrate (see Materials and Methods). Here we found that the Gpx4 activity of MEL cells transfected with the anti-Gpx4 antisense plasmid was about 60% lower than that of the mock-transfected cells (Figure 2c). Taken together the data shown in Figure 2 indicated that expression of the *Gpx4* gene was strongly reduced but not abolished in siRNA transfected cells. To test whether expression silencing of Gpx4 impacts basic cellular functions we compared the cell division kinetics of mock-transfected and Gpx4 siRNA transfected cells (Figure 2d). When equal numbers of cells (10^4^ cells/mL) were seeded into the culture dishes we did not find significant differences between the two cell types after 24 h of incubation. After 48 h the cell division rate of Gpx4 RNAi cells was slightly increased compared to the corresponding control transfectant (MOCK RNAi) (*p* = 0.087). After 72 and 96 h, the division rate of Gpx4 RNAi cells was significantly higher compared to MOCK RNAi cells. Moreover, the cell doubling time of Gpx4-deficient MEL cells (T_d_ = 15.05 ± 0.21) was significantly (*p =* 0.0006) lower when compared with that of mock-transfected MEL cells (T_d_ = 18.76 ± 0.38). On the other hand, we did not observe obvious signs of premature cell death (Trypan blue staining, data not shown) and these findings were somewhat surprising in the lights of the previous reports on the cell protecting character of Gpx4 in mouse erythroid precursors [21].

### 2.4. Erythropoiesis Can Be Induced in Mock-Transfected and Gpx4 siRNA Transfected MEL Cells

**Figure 3:**
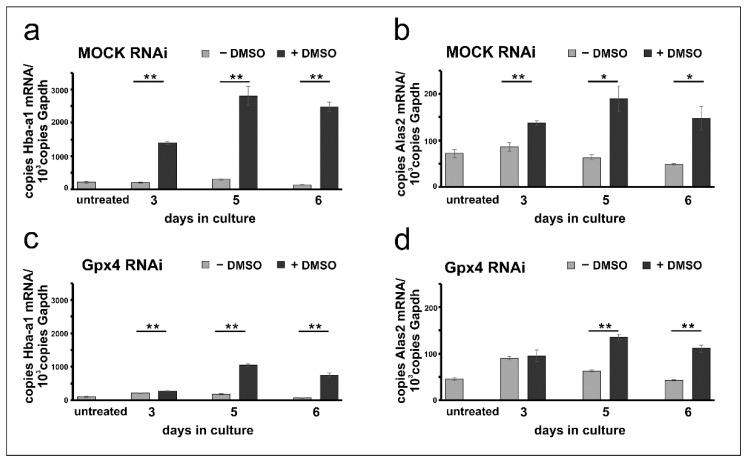
DMSO induces erythroid differentiation in mock-transfected and Gpx4 siRNA transfected MEL cells. Total RNA was extracted from untreated and DMSO-treated MEL cell transfectants at different days of the culture period. The steady-state concentrations of Hba-a1, Alas2, and Gapdh mRNAs were quantified by qRT-PCR using the specified amplification primers as described in the Material and Methods section. Each sample was quantified in triplicates. (**a**) Expression of Hba-a1 mRNA in MOCK RNAi MEL cells. (**b**) Expression of Alas2 mRNA in MOCK RNAi MEL cells. (**c**) Expression of Hba-a1 mRNA in Gpx4 RNAi MEL cells. (**d**) Expression of Alas2 mRNA in Gpx4 RNAi MEL cells. Significances were calculated using the Student’s *t*-test, * *p* < 0.05, ** *p* < 0.005.

Erythropoiesis can be induced in resting MEL cells when the cells are stimulated with DMSO and erythroid differentiation can be followed by quantification of the expression of several erythroid-specific genes [26,28,30]. For our experiments we quantified the steady state concentrations of the mRNAs encoding for hemoglobin a-a1 (Hba-a1) and for the 5-aminolevulinic acid synthase 2 (Alas2), the key enzyme of heme biosynthesis, at different time-points of the differentiation process. From Figure 3a it can be seen that in mock-transfected cells the expression of the Hba-a1 mRNA is elevated following DMSO stimulation. In contrast, Hba-a1 expression remained minimal in cells cultured in the absence of DMSO and there were highly significant differences between DMSO-treated and untreated mock-cells at all time points of the differentiation period. Similar Hba-a1 expression kinetics were previously reported for wildtype Mel cells [24,27,32]. Next, we explored the expression profiles for Alas2 and observed similar kinetics. Expression of this mRNA was induced by DMSO and significant differences to unstimulated cells were observed (Figure 3b). 

To explore whether erythropoiesis can also be induced in Gpx4-deficient MEL cells similar differentiation studies were carried out with cells transfected with the anti-Gpx4 antisense plasmid. Here we found that expression of the Hba-a1 mRNA is also induced by DMSO and significant differences to untreated (- DMSO) cells were observed at all time points of the differentiation period. Interestingly, at day 3 of the differentiation period the degree of DMSO-dependent upregulation of Hba-a1 expression was less pronounced for Gpx4 deficient MEL cells (Figure 3c) when compared with the corresponding mock-controls (Figure 3a). In fact, for the mock-cells we observed a 7-fold higher (720%) steady state concentration of the Hba-a1 mRNA in DMSO-treated cells when compared with untreated mock-controls (Figure 3a, day 3). In contrast, for Gpx4-deficient MEL cells the Hba-a1 mRNA concentration was only increased by about 30%. Similarly, differential expression kinetics were observed when Alas2 mRNA was profiled. Here again, significant differences were detected at day 5 and day 6 of the differentiation period when DMSO-treated cells were compared with untreated cells (Figure 3d). In contrast, no difference between DMSO-treated and untreated cells was found on day 3. 

### 2.5. Stable Knockdown of Gpx4 Expression Delays DMSO-Induced Erythroid Differentiation

**Figure 4:**
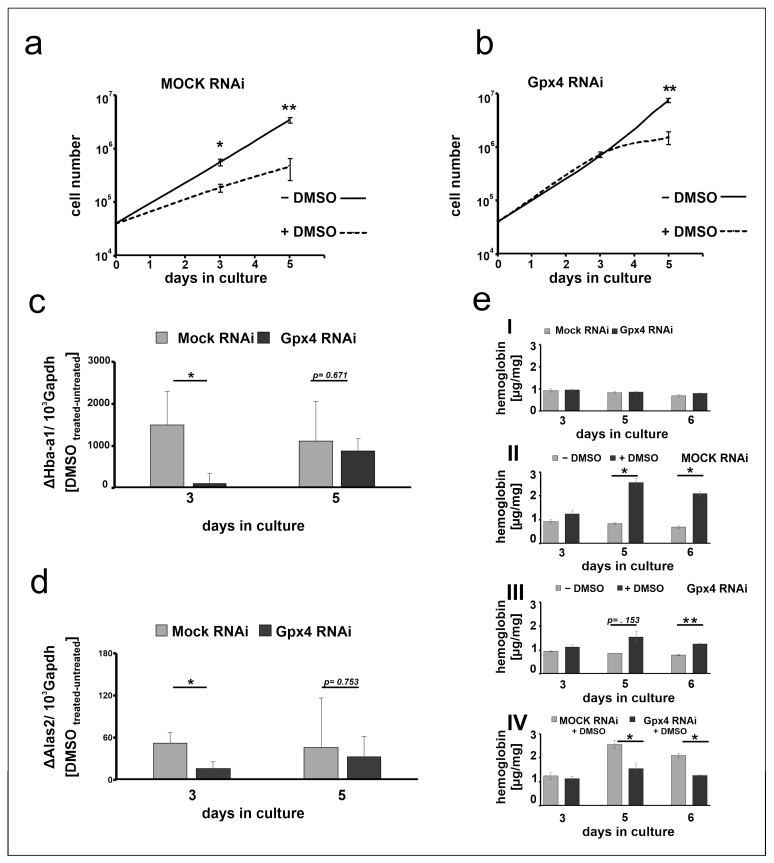
Stable knockdown of Gpx4 expression retards DMSO-induced erythroid differentiation. (**a**,**b**) MEL cell division rate of stably transfected RNAi MEL cells. MEL cells, stably transfected with a Gpx4 siRNA plasmid (Gpx4 RNAi) (**b**) or with a corresponding control vector (MOCK RNAi) (**a**) were seeded into culture dishes (10^4^ cells/mL) in triplicates and cultured at 37 °C for 5 days in the absence (− DMSO) or in the presence of DMSO (+ DMSO) as described in Material and Methods. Cells were counted on day 3 and day 5. The results are presented as cumulative cell number *versus* days in culture. Significances were calculated using the Student’s *t*-test, n = 3, * *p* < 0.05, ** *p* < 0.005. (**c**) Hba-a1 mRNA and (**d**) Alas2 mRNA expression stimulation after DMSO treatment of stable transfected RNAi MEL cells. Total RNA was extracted from untreated and DMSO-treated MEL cell transfectants at different days of the culture period. The steady-state concentrations of Hba-a1 (**c**), Alas2 (**d**) related to Gapdh mRNAs were quantified by qRT-PCR using the specified amplification primers as described in the Material and Methods section. (**e**) Hemoglobin synthesis in stably transfected RNAi MEL cells without (− DMSO) or with DMSO (+ DMSO) stimulation. Intracellular hemoglobin levels at different days of the culture period were quantified as described in Material and Methods. (**eI**) Hemoglobin content of unstimulated MEL transfectants. (**eII**) Hemoglobin content of mock- transfected MEL cells (MOCK RNAi) after DMSO treatment, (**eIII**) Hemoglobin content of Gpx4 siRNA transfected MEL cells (Gpx4 RNAi) after DMSO treatment, (**eIV**) Hemoglobin content of DMSO treated Gpx4 deficient MEL cells (Gpx4 RNAi) and the corresponding control transfectant (MOCK RNAi). Significances were calculated using the Student’s *t*-test, * *p* < 0.05, ** *p* < 0.005.

The data shown in Figure 3 suggest that at day 3 of the differentiation period the steady state concentrations of the Hba-a1 and Alas2 mRNA are significantly elevated in DMSO-treated mock-transfected MEL cells when compared with untreated controls (Figure 3a,b). In contrast, in Gpx4-deficient cells the degree of elevation is strongly reduced when Hba-a1 mRNA is used as readout parameter (Figure 3c) and no significant difference is seen for Alas2 (Figure 3d). These data suggest that in Gpx4-deficient MEL cells DMSO induced erythropoiesis may be delayed. To obtain additional evidence for the delay of erythroid differentiation in Gpx4-deficient MEL cells, we next explored the impact of DMSO on cell proliferation of Gpx4-sufficient and Gpx4-deficient MEL cells. In principle, erythropoiesis involves two counterbalanced processes: (i) Upregulation of functional specialization that is paralleled by a loss in functional multiplicity. (ii) Downregulation of the proliferation potency [28]. In other words, cells with a high proliferation capacity exhibit a low degree of differentiation. *Vice versa*, differentiated cells have a reduced proliferation potential. To explore whether Gpx4 expression silencing impacts the proliferative potential of MEL cells during erythroid differentiation, stably transfected Gpx4 RNAi MEL cells and the corresponding control transfectant (MOCK RNAi) were cultured in suspension for 5 days. When the MEL transfectants were cultured in the absence of DMSO, they divided with a certain rate (Figure 4a,b). In the presence of DMSO the cell division rate was significantly reduced (Figure 4a, dotted line). This observation is consistent with the higher degree of differentiation of the cells. In contrast, when Gpx4 RNAi MEL cells were taken through the same experimental protocol we did not observe impairment of cell division at day 3 of the culture period. From this data one may conclude that there is no major alteration in the degree of erythroid differentiation between Gpx4-sufficient and Gpx4-deficient cells at this time point. However, at later time points of the culturing period (5 days of DMSO treatment) we observed significant differences in the cell division characteristics (Figure 4b, dotted line). The impaired proliferation potential of DMSO-treated Gpx4-deficient cells (compared with untreated Gpx4-deficient cells) suggested a higher degree of erythroid differentiation of the untreated cells. Thus, on the basis of these proliferation data one might conclude that Gpx4-deficiency delays DMSO-induced erythroid differentiation.

To obtained more direct evidence for this conclusion we next quantified the DMSO sensitivity of mock-transfected MEL cells with that of Gpx4-deficient cells employing the steady state concentration of the Hba-a1 and Alas2 mRNA as readout parameter. For this purpose, we first subtracted from the mRNA copy numbers of DMSO-treated mock-transfected cells at day 3 of the differentiation period the corresponding value of untreated mock transfectants. The resulting difference appears to be a suitable measure for the DMSO sensitivity of these cells. Then, we repeated this procedure for Gpx4-deficient cells at day 3 of the differentiation period and compared the resulting differences. From Figure 4c,d it can be seen that at day 3 of the differentiation period mock-transfected MEL cells were more sensitive for DMSO-induced Hba-a1 and Alas2 expression than Gpx4-deficient cells. In contrast, on day 5 of the differentiation period, there was no significant difference in the DMSO sensitivity of the two cell types anymore. These data are consistent with our previous conclusion that Gpx4-deficiency delays in vitro erythropoiesis of MEL cells by reducing their sensitivity for DMSO. 

To further support this conclusion we quantified the hemoglobin content of DMSO-stimulated mock-transfected MEL cells with the corresponding Gpx4-deficient counterparts. As indicated in Figure 4(eI) unstimulated MEL cells did only express small amounts of hemoglobin regardless of whether the expression of Gpx4 was compromised or not. When mock-transfected cells were stimulated with DMSO a time-dependent increase in hemoglobin expression was observed (Figure 4(eII)) and significant differences between DMSO-treated and untreated cells were detected at day 5 and day 6 of the differentiation period. In contrast, when Gpx4-deficient cells were taken through the same experimental protocol we did not see significant differences between DMSO-treated and untreated cells at day 5 of the differentiation period (Figure 4(eIII)). Compared with mock-transfected cells the hemoglobin content was significantly lower after DMSO stimulation (Figure 4(eIV)). These data support our conclusion that partial Gpx4 deficiency delays but did not abolish in vitro erythropoiesis in this cellular in vitro model. 

**Figure 5:**
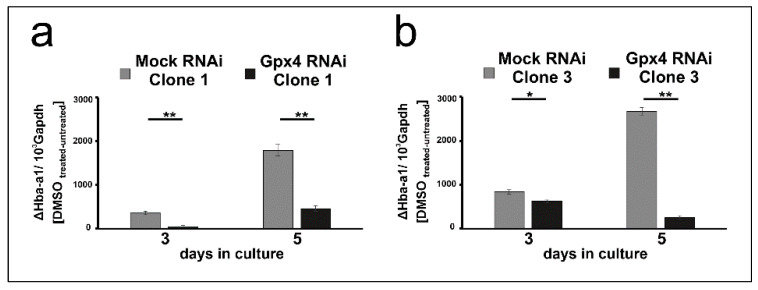
Delay of DMSO-induced erythroid differentiation in two additional clones of Gpx4 deficient MEL cells. MEL cells, stably transfected with a Gpx4 siRNA plasmid (Gpx4 RNAi) or with a corresponding control vector (MOCK RNAi) were seeded in culture dishes (10^4^ cells/mL) and cultured at 37 °C for 5 days without or with 2% DMSO (see Material and Methods). Total RNA was extracted from untreated and DMSO-treated MEL cell transfectants at different days of culturing period. The steady-state concentrations of Hba-a1mRNA and Gapdh mRNA were quantified by qRT-PCR using specific amplification primers. (**a**,**b**) Different individual stably transfected RNAi MEL cell clones. Significances were calculated using the Student’s *t*-test, * *p* < 0.05, ** *p* < 0.005.

To put our conclusions on a broader experimental basis, the in vitro differentiation experiments were repeated with two additional clones Gpx4 RNAi transfectants (clones 1 and 3 of Figure 1) and the steady state concentrations of Hba-a1 mRNA on day 3 and day 5 of DMSO treatment were quantified as readout parameter. To compare the DMSO sensitivity of the mock transfectants with that of the Gpx4 deficient MEL cells, the Hba-a1mRNA expression of the untreated cell was subtracted from the corresponding value of the DMSO treated cell. From Figure 5a,b it can be seen that on day 3 and day 5 of the differentiation period erythroid differentiation was compromised in the additional clones of Gpx4-RNAi transfectants. These data are consistent with our previous conclusion that Gpx4-deficiency delays in vitro erythropoiesis of MEL cells.

### 2.6. Delay of DMSO Induced In Vitro Erythropoiesis in MEL Cells Caused by Gpx4 Deficiency Was Partially Rescued by Ebselen

Gpx4 is an antioxidative enzyme and anemia induced by in vivo Gpx4 expression silencing in hematopoietic cells [21] can be partially rescued by vitamin E supplementation. On the other hand, in vitro erythroid differentiation studies of human CD34^+^ cells demonstrate a role for Gpx4 in the enucleation of human erythroblasts (late phase of erythropoiesis). Gpx4 knockdown strongly impaired enucleation but this effect cannot be reversed by the addition of alpha-tocopherol [33]. Here we explored first whether addition of alpha-tocopherol can also rescue the compromised DMSO-induced erythropoiesis in Gpx4 siRNA transfected MEL cells. For this purpose, we induced erythropoiesis with DMSO in siRNA transfected MEL cells in the presence or absence of 100 µM alpha-tocopherol. As differentiation marker we quantified the steady state levels of Hba-a1 and Alas2 mRNAs on days 3 and 5 after DMSO addition using qRT-PCR. Under these conditions, we did not observe any rescue effect (data not shown).

**Figure 6:**
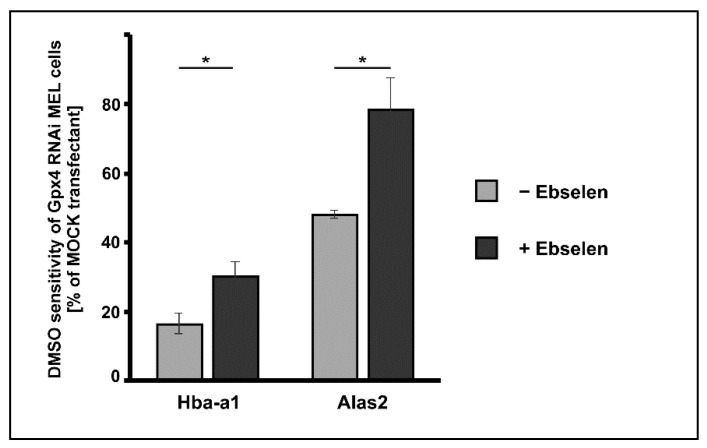
Delay of DMSO-induced erythroid differentiation in Gpx4 deficient MEL cells was partially rescued by the seleno-organic compound ebselen that functions as Gpx mimetic. MEL cells, which were stably transfected with a Gpx4 siRNA plasmid (Gpx4 RNAi) or with a corresponding control vector (MOCK RNAi) were seeded in culture dishes (10^4^ cells/mL) and cultured in the absence or presence of 5 µM ebselen. Cells were cultured at 37 °C for 3 days without or with 2% DMSO and total RNA was extracted. The steady-state concentrations of Hba-a1 mRNA, Alas2 mRNA and Gapdh mRNA were quantified by qRT-PCR using specific amplification primers. DMSO sensitivity in the different incubation samples was determined and the DMSO sensitivity of the Gpx4 RNAi transfectant was shown relative to that of the MOCK transfectant (see Material and Methods). Significances were calculated using the Student’s *t*-test, * *p* < 0.05.

Ebselen is a seleno-organic compound that functions as Gpx-mimetic. It reduces lipid hydroperoxides to the corresponding alcohols at the expense of reduced glutathione [34]. However, the catalytic efficiency of this non-enzymatic catalyst is much lower than that of Gpx-isoforms. We employed this seleno-organic compound to perform rescue experiments and tested whether addition of ebselen restores the impaired DMSO-induced in vitro erythropoiesis in Gpx4 deficient MEL cells. For this purpose, we first tested the cell toxicity of ebselen for Gpx4 RNAi- and MOCK-transfected MEL cells and found that both cell types tolerated 5 µM ebselen without major signs of cell death. Next, we induced erythropoiesis with DMSO in Gpx4 RNAi- and MOCK-transfected MEL cells in the presence or absence of 5 µM ebselen. As differentiation marker we quantified the steady state levels of Hba-a1 and Alas2 mRNA on days 3 after DMSO addition using qRT-PCR. Under these experimental conditions, the DMSO sensitivity of the Gpx4 RNAi transfectant was significantly higher in the presence of ebselen but we did not observe complete rescue of the Gpx4-deficient phenotype (Figure 6). To improve the rescue effect of ebselen we performed similar experiments at increased ebselen concentrations (50 µM) but observed significant cell toxicity for both cell types.

### 2.7. Heterozygous Expression of Catalytically Inactive Gpx4 in Mice Does Not Induce a Defective Erythropoietic Phenotype

**Figure 7:**
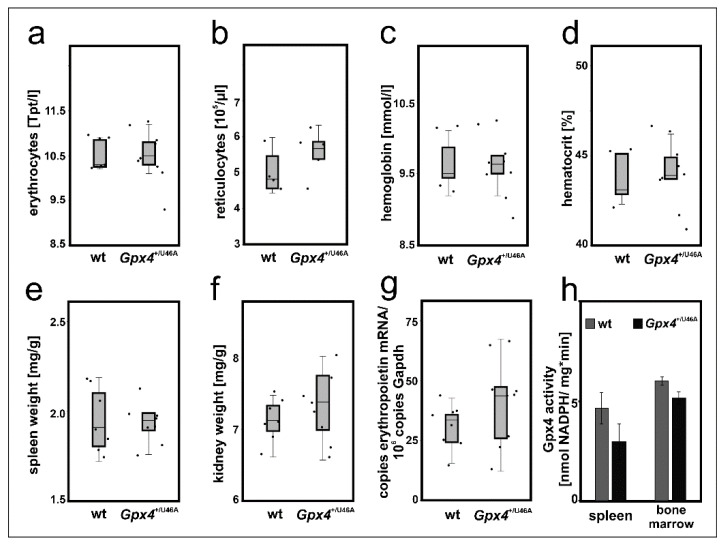
Heterozygous expression of catalytically inactive Gpx4 in mice does not induce a defective erythropoietic phenotype. 200 µL blood was withdrawn from male Sec46Ala Gpx4 mutant (*Gpx4*^+/U46A^) and corresponding wild type (wt) mice. For blood removal mice were sacrificed by cervical dislocation after isoflurane anesthesia and blood was collected by cardiac puncture. Coagulation was inhibited by EDTA and the basic red blood cell parameters were determined as described in Material and Methods. (**a**) erythrocyte count, (**b**) reticulocyte counts, (**c**) hemoglobin, (**d**) hematocrit. n = 5–8 animals were analyzed. The wet weights of spleens and kidneys of male Sec46Ala Gpx4 mutant (*Gpx4*^+/U46A^) and corresponding wild type (wt) mice were determined and the bodyweight/organ weight ratios were calculated. (**e**) spleen/body weight ratios, (**f**) and kidney/body weight ratios. n = 7–8 animals were analyzed. (**g**) Renal expression of erythropoietin (Epo) mRNA. Total RNA was extracted from kidneys of wild type (wt) and Sec46Ala Gpx4 mutant (*Gpx4*^+/U46A^) mice. Epo mRNA expression levels were quantified by qRT-PCR (see “Material and Methods”). For these experiments, n = 8 animals were sacrificed and each sample was quantified in triplicate. Significances were calculated using the Mann-Whitney U test and *p*-values < 0.05 were considered statistically significant. (**h**) Gpx4 activity assays. Spleen and bone marrow cells were homogenized, the 10,000 g lysate supernatant was prepared and the Gpx4 activity was assayed using RP-HPLC purified phosphadidylcholine hydroperoxide as substrate. Significances were calculated using the Student’s *t*-test. Two animals of each genotype were used and each sample was quantified in triplicates.

The cellular differentiation experiments suggested that a 50–60% reduction of Gpx4 expression in MEL cells delayed DMSO-induced in vitro erythropoiesis (Figure 3 and Figure 4). To test whether such retardation can also be observed in vivo we employed heterozygous *Gpx4* knock-in mice, in which the *Gpx4* gene locus was modified in such a way, that the animals express heterozygously the catalytically silent Sec46Ala Gpx4 mutant (*Gpx4*^+/Sec46Ala^). Homozygous *Gpx4*^Sec46Ala/Sec46Ala^ mice do not survive day 6 of embryo development but heterozygous allele carriers are viable, reproduce and do not show obvious phenotypical alterations unless stimulated otherwise [16]. To explore whether our genetic manipulation induced defective erythropoiesis we compared the basic erythrocyte parameters of these mice with the corresponding values of control animals with a similar genetic background (Figure 7a–d) but did not detect significant differences.

For independent support of this conclusion we compared the relative organ weights of spleen and kidney between the two genotypes. In mice, stress erythropoiesis proceeds in the spleen [35] and if permanent Gpx4 deficiency challenges the erythropoietic system a compensatory increase in the relative spleen size would be expected. Unfortunately, we did not observe a significant difference in the relative spleen size between *Gpx4*^+/Sec46Ala^ mice and corresponding controls (Figure 7e). Another possible compensation mechanism would be an increased renal erythropoietin synthesis in *Gpx4*^+/Sec46Ala^ mice. To test this option we first compared the relative kidney weights of wildtype and *Gpx4*^+/Sec46Ala^ mice but did not find significant differences (Figure 7f). Next, we quantified renal erythropoietin mRNA concentration but here again, we did not detect significant differences (Figure 7g). Finally, we assayed the Gpx4 activity in bone marrow cells and spleen of *Gpx4*^+/Sec46Ala^ mice and compared the data with the corresponding values obtained for wild-type mice. Here we neither observed significant differences between the two genotypes (Figure 7h). Taken together, these data did not provide any evidence for a compromised erythropoiesis in *Gpx4*^+/Sec46Ala^ mice.

### 2.8. Heterozygous Expression of Catalytically Inactive Gpx4 in Mice Does Not Impact Ex Vivo Erythropoiesis of Embryonic Liver Derived Erythroblasts

**Figure 8:**
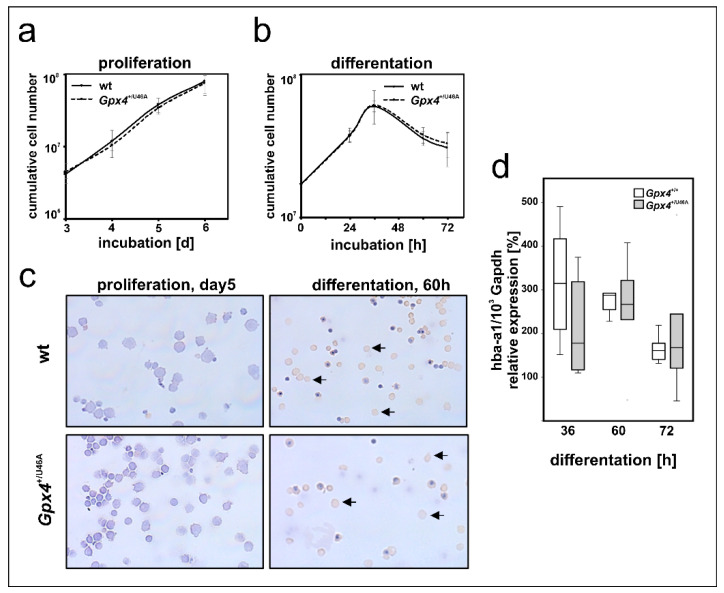
Heterozygous expression of a catalytically inactive Gpx4 in mice does not impact ex vivo erythropoiesis of embryonic liver derived erythroblasts. Fetal livers of day 12.5 mouse embryos (wild type and *Gpx4*^+/U46A^) were prepared, homogenized and primary erythroblasts were expanded for 5 days in STEMPRO^TM^-34 SFM medium containing 50 ng/mL recombinant murine stem cell factor, 1 U/mL human erythropoietin-alpha, and 1 µM dexamethasone. Terminal erythroid differentiation was induced in continuously proliferating erythroblasts by exposure to 5 U/mL erythropoietin-alpha and 0.5 mg/mL iron loaded human transferrin as described in Material and Methods. (**a**) Cumulative cell number of proliferating erythroblasts derived from *Gpx4*^+/U46A^ and the corresponding wild-type controls (n = 3). (**b**) Cumulative cell number after induction of terminal erythroid differentiation (n = 3). (**c**) Morphological and histochemical analysis of proliferating and differentiated wild type (upper panels) and *Gpx4*^+/U46A^ (lower panels) mouse erythroblast. Proliferating erythroblasts (5 days) and erythroblasts that have been differentiated for 60 h in the presence of erythropoietin were HE stained and subsequently counterstained stained with neutralized benzidine to detect hemoglobin expression (arrows). (**d**) Hba-a1 mRNA expression during terminal differentiation in wildtype (*Gpx4*^+/+^) and Gpx4 deficient (*Gpx4*^+/U46A^) cells. Total RNA was extracted at different time points of the culturing period. The steady-state concentrations of Hba-a1 mRNA was related to Gapdh mRNA as described in the Material and Methods section. n = 5. Significances were calculated using the Mann-Whitney U test and *p*-values < 0.05 were considered statistically significant.

In early stages of mammalian embryogenesis, the liver plays a major role in erythropoiesis [36,37]. Primary mouse erythroblasts can be prepared from fetal mouse liver and erythropoiesis can be induced in these cells by the addition of erythropoietin (EPO) [38,39]. Since our in vivo studies with *Gpx4*^+/Sec46Ala^ mice did not support our conclusion (Gpx4 deficiency delays erythropoiesis) drawn from our in vitro data we carried out additional ex vivo experiments to explore the possibility that ex vivo erythropoiesis of primary mouse erythroblasts prepared from *Gpx4*^+/Sec46Ala^ mice might be compromised. For this purpose we prepared hepatocytes from embryos of wildtype and *Gpx4*^+/Sec46Ala^ mice, expanded the primary mouse erythroblasts, and induced in vitro erythropoiesis by the addition of EPO [38,39]. As readout parameters for erythroid differentiation we compared the proliferation kinetics of the two cell types and the expression of Hba-a1 as erythroid marker (Figure 8). Unfortunately, here we neither observe significant differences between the two genotypes.

## 3. Discussion

### 3.1. Gpx4 Isoforms Are Expressed in MEL Cells

To contribute to the discussion on the potential role of Gpx4 isoforms in erythropoiesis we selected the frequently employed in vitro erythropoiesis model of cultured MEL cells and explored whether expression silencing of this enzyme impacts the differentiation kinetic of these cells. To find out whether these cells are a suitable model for our experimental strategy we first quantified the expression of Gpx4 on mRNA, protein, and activity levels. As indicated in Figure 1a cGpx4 mRNA is the most abundant isoform. The expression levels of the different Gpx4-isoforms (cGpx4, mGpx4, and nGpx4) in MEL cells are comparable to those in mouse somatic tissues [16]. 

### 3.2. Gpx4 and Erythropoiesis

When the *Gpx4* gene was specifically deleted in hematopoietic cells mice develop anemia and mechanistic studies employing these tissue specific knockout mice suggested that Gpx4 is essential for preventing receptor-interacting protein 3 (RIP3)-dependent necroptosis in erythroid precursor cells [21]. In fact, disruption of the *Gpx4* gene induces functional inactivation of caspase 8 by glutathionylation. This post-translational modification of caspase 8 induces necroptosis, which proceeds independently of tumor necrosis factor α activation. Genetic ablation of Rip3 normalizes erythropoiesis and prevents anemia. These results demonstrate that erythroid-specific *Gpx4* deficiency activates RIP3-dependent necroptosis in erythroid precursor cells [21].

In a more recent study the potential role of Gpx4 in the later stage of erythropoiesis, in particular in reticulocyte-erythrocyte transition was explored [29]. Quantification of the basic erythrocyte parameters in the peripheral blood and the bone marrow of chimeric mice with *Gpx4* deficiency in erythropoietic cells revealed anemia with a compensatory increase of early erythroid precursors and reticulocytes. Additional dietary depletion of vitamin E aggravated the anemic phenotype. Interestingly, peripheral reticulocytes failed to mature normally but the cells accumulated large autophagosomes with engulfed mitochondria. Moreover, *Gpx4*-deficiency induced systemic hepatic iron overload, increased iron demand in the erythropoietic system, and strongly elevated erythropoietin levels in blood plasma. From these data the authors concluded that *Gpx4* deficiency disturbs reticulocyte-erythrocyte transition, which constitutes the last step of erythropoiesis [29]. In addition to the role of Gpx4 in murine erythropoiesis, a role during humane erythroid maturation has also been demonstrated. Inhibition of GPX4 was shown to impair enucleation of human erythroblasts in a ferroptosis-, mitophagy-, and necroptosis-independent manner [33].

Taken together these studies suggested a role of Gpx4 in early [21] and late erythropoiesis [29,33]. To contribute to the discussion on the potential role of Gpx4 in erythropoiesis we employed the frequently used MEL cell system. This cellular in vitro model mirrors the second phase of erythroid differentiation, which involves the maturation of nucleated proerythroblasts to erythroblasts [1,28]. This phase is characterized by hemoglobin biosynthesis, a decrease in cell size and nuclear condensation. Resting MEL cells do not express hemoglobin but stimulation with DMSO induces the expression of erythroid specific genes such as hemoglobin and δ-ALA synthase [28,30]. To induce Gpx4 deficiency we stably transfected MEL cells with a RNAi construct and this technique induced a more than 50% reduction of Gpx4 expression (Figure 1). Interestingly, the transfection procedure did not induce major alterations in basic cell characteristics. These Gpx4-deficient cells were viable and showed similar growth kinetics as corresponding mock-transfected controls (Figure 2d). When erythropoiesis was induced by incubating the cells with DMSO we observed a temporal delay of erythropoiesis and these results suggested that Gpx4 may also be important for the 2nd phase of erythropoiesis. Together with the above mentioned in vivo studies [21,29] our in vitro data suggest a role of Gpx4 in all three stages of erythropoiesis. 

The molecular basis for the Gpx4-deficiency induced delay of in vitro erythropoiesis has not been explored in this study. Gpx4 expression silencing is likely to impact the redox state of the MEL cells and thus may modify the expression of redox regulated genes. Such genes have been implicated in erythropoiesis [28,40,41] and their catalytic activity might be modulated by Gpx4 expression silencing.

As many permanent cell lines MEL cells are cancer cells. Nevertheless, they have frequently been used as model systems for in vitro erythropoiesis since they adequately mirror the second phase of mouse erythropoiesis. We cannot exclude that the retardation effect we observed may be related cancer character of these cells and this is a limitation of this study. However, similar limitations are also applicable to any other studies using cancer cell lines including previous erythropoiesis studies with MEL cells [28,30].

### 3.3. Heterozygous Expression of a Catalytically Inactive Gpx4 Mutant Does Not Compromise In Vivo Erythropoiesis in Mice

To translate our in vitro findings into the in vivo situation we explored *Gpx4*^+/U46A^ mice. These knock-in mice express heterozygously the catalytically silent Sec46Ala Gpx4 mutant. The animals express significantly lower amounts of catalytically active Gpx4 and comparative activity assays with testis and brain indicated a 50% reduction of specific Gpx4 activity [16]. A similar reduction in the catalytic activity was observed when Gpx4-RNAi-transfected MEL cells were compared with corresponding mock-transfectants (Figure 2c). From this activity comparison we hypothesized that the *Gpx4*^+/U46A^ knock-in mice might also exhibit a defective erythropoietic phenotype. To test this hypothesis, we compared the basic erythrocyte parameters of *Gpx4*^+/U46A^ mice and corresponding wild type controls (Figure 7a–d) but did not observe significant differences. To exclude major compensation reactions, we also compared spleen weights (upregulated extramedullary erythropoiesis) and kidney weights (elevated erythropoietin production) but neither observed significant differences between *Gpx4*^+/U46A^ mice and wildtype controls (Figure 7e,f). Moreover, we found that renal erythropoietin synthesis was not upregulated in *Gpx4*^+/U46A^ mice (Figure 7e). Taken together, these data suggest that heterozygous expression of a catalytically silent Gpx4 mutant did not compromise in vivo erythropoiesis. It looks like that this genetic manipulation may not be severe enough to induce defective red cell development in either of its three stages.

## 4. Materials and Methods

### 4.1. Chemicals and Reagents

The chemicals were from the following sources: DMSO from AppliChem (Darmstadt, Germany), Bradford Reagenz from Roth (Karlsruhe, Germany), glutathione, glutathione reductase, tetramethylbenzidine, hemoglobin, NADPH and α-tocopherol from Sigma-Aldrich (Steinheim, Germany), ebselen from Rhone-Poulenc Rorer GmbH (Cologne, Germany) and isoflurane from AbbVie (Ludwigshafen, Germany). Oligonucleotide synthesis was performed at BioTez (Berlin, Germany). Other chemicals were purchased from Invitrogen (Darmstadt, Germany).

### 4.2. Animals, Breeding, and Isolation of Spleen and Bone Marrow

All the mice were bred and maintained in a specific pathogen-free (SPF) animal facility, on 12 h/12 h LD cycle, with food and water ad libitum. Procedures were performed according to the EU Directive 2010/63/EU, Federation of Laboratory Animal Science Associations and the local guidelines. All animal experiments were performed in compliance with the German animal welfare law and have been approved by the institutional committee on animal experimentation. *Gpx4*^+/U46A^ mice were created as described before [16] and corresponding wildtype control animals were outbred by mating heterozygous allele carriers. Separate colonies of *Gpx4*^+/U46A^ mice and outbred wild-type controls were maintained. All individuals were genotyped.

### 4.3. PCR Genotyping

Genomic DNA was prepared from ear punch using Invisorb Spin Tissue Mini Kit (Invisorb, Berlin, Germany) and genomic PCR was carried out with MyTaq^TM^Red Mix (BIOLINE, Heidelberg, Germany) system. Primers used for analyzing the *Gpx4* gene locus were as follows: up- 5′- GACAGATGGCTCTGGACCTGGGTG-3′, do-: 5′-TAATCTGGCG TGGTAGGGGCA GAC-3′. The wildtype allele showed up as a 412 bp fragment but the *Sec46Ala* mutant allele gave an amplification product of higher molecular weight (587 bp). The higher molecular weight was due to a residual LoxP/FRT sequence in the genome after deletion of the neo-cassette. Heterozygous allele carriers showed two bands in PCR.

### 4.4. MEL Cell Culture

Murine erythroleukemia cells (MEL-745A cl.DS19) were obtained from the German Collection of Microorganisms and Cell Cultures (Leibniz Institut, Braunschweig, Germany) and clone 19 is described to be DMSO sensitive, resulting in erythroid differentiation. Cells were grown in RPMI-medium (PAN BIOTECH, Aidenbach, Germany) containing 10% fetal calf serum (PAN BIOTECH) at 37 °C with 5% CO_2_. 

### 4.5. Antisense Transfection and Selection Procedure

The siRNA plasmids were kindly provided by Dr. Nicolai Savaskan [32]. Briefly, for Gpx4 mRNA silencing the pSuper plasmid vector system was used (NKI, Amsterdam, the Netherlands). The target sequence for murine GPx4 5′-TGGTCTGCCTGG ATA AGT A-3′ was cloned into the pSuper-GFP (Gpx4 siRNA) vector according to the manufacturer’s recommendations (NKI) as described in [42]. As control empty pSuper-GFP vector was used (MOCK siRNA). For stable transfection, MEL cells were transfected with either the Gpx4 si RNA plasmid or the corresponding control plasmid using TransIT-LT1 Transfection Reagent (Mirus, Madison, WI, USA) according to the manufacturer’s recommendations. Individual clones were selected with 400 µg/mL geneticin (G418, Roth) and the degree of Gpx4 mRNA silencing was quantified by quantitative real-time PCR (see Section 4.7) Three representative subclones of Gpx4 siRNA and mock-transfected MEL cells were selected for further analysis. The cell viability was routinely examined by the trypan blue staining. 

### 4.6. DMSO Induced Erythroid Differentiation in Stable MEL Cell Transfectants

Stable MEL cell transfectants were seeded at 10^4^ cell/mL. Erythroid differentiation of the cell culture was induced by adding DMSO to the final concentration of 2%. Stimulated and untreated control cells were cultured for up to 6 days. Cells were kept in a concentration of 0.5 × 10^6^ cells/mL. The cell viability was routinely examined by the trypan blue staining. Aliquots were harvested for RNA isolation (see Section 4.7) and hemoglobin determination (see Section 4.8).

For growth analysis cells were counted every day and doubling time was calculated in the exponential phase of the growth curves using the following formula: Td = T_x_ × ln (2)/ln (N_x_/N_0_), were Td—cell population doubling time, T_x_—cultivation time, N_x_—number of cells at T_x_ time point, and N_0_—the initial number of cells [43]. For rescue experiments we added 100 µM α-tocopherol or 5 µM ebselen to the culture medium before erythroid differentiation was induced by adding DMSO to the final concentration of 2%. The steady-state concentrations of Hba-a1 mRNA, Alas2 mRNA and Gapdh mRNA were quantified by qRT-PCR using specific amplification primers (Table 1). For the determination of the DMSO sensitivity of the Gpx4 RNAi transfectant, the normalized expression of Hba-a1 and Alas2 in the DMSO-untreated sample was subtracted from the DMSO-treated sample and then related to the corresponding mock transfectant.

### 4.7. RNA Isolation and Quantitative Real-Time PCR

Total RNA was extracted using the NucleoSpin RNA Plus (Macherey-Nagel, Düren, Germany). Synthesis of cDNAs was performed with 0.1–2 µg of the total RNA preparations using oligo (dT) primers and Tetro Reverse Transcriptase (BIOLINE, Luckenwalde, Germany). Quantitative real-time PCR (RT-PCR) was carried out with a Rotor Gene 3000 (Corbett Research, Sydney, Australia) using the SensiFast SYBR PCR Kit (BIOLINE). The following primer sets were employed to quantify the Gapdh, mGpx4, m + cGpx4, nGpx4, Epo, Hba-a1, Alas2 (Table 1). The Rotor-Gene Q software was used to analyze the PCR raw data. Specific amplicons were prepared as external amplification standards for all target and reference (Gapdh) genes and this procedure allowed exact quantification of the mRNA copy numbers. Gapdh mRNA was used as an internal amplification standard and expression of the target genes was normalized to the Gapdh content. Analyses were performed at least in triplicate and mean ± standard deviations are given.

### 4.8. Photometric Hemoglobin Determination

Hemoglobin concentrations in the MEL cell lysates were measured by the tetramethylbenzidine assay [44]. Small amounts of cell culture were removed to different time points of incubation. Pellets of MEL cells were lysed by the addition of 5% acetic acid and centrifuged at 10,000× *g* for 10 min. The supernatant was transferred to a new tube and total protein concentration was determined by the Bradford method [45]. Tetramethylbenzidine assay was adapted to 96 well plates and the absorbance was measured at 600 nm. Values obtained from triplicates were normalized to the total protein content of the cell lysate. Hemoglobin concentration was determined by comparing the absorbance values to a standard curve prepared by similar incubation of pure human hemoglobin solutions.

### 4.9. Immunoblotting

Protein extracts were prepared from stable MEL cell RNAi transfectants (mock-transfected and Gpx4 RNAi). Aliquots with an equal amount of total protein (100 µg) were separated on 12.5% sodium dodecyl sulfate-polyacrylamide gel electrophoresis gels. The proteins were transferred to a nitrocellulose membrane (Amersham, Buckinghamshire, UK). The membrane was blocked with Blotting Grade Blocker (Bio-Rad, Feldkirchen, Germany) and incubated overnight with the monoclonal anti-human Gpx4 antibody (#MABF1969, Merck, Darmstadt, Germany) [31], or anti-GAPDH (Sigma, Taufkirchen, Germany). The membrane was washed with PBS/0.3% Tween 20 and incubated with secondary antibodies (anti-mouse: Sigma or anti-rabbit: Santa Cruz, Heidelberg, Germany) for 30 min. Blots were developed with an ECL kit (PerkinElmer, Rodgau, Germany).

### 4.10. Gpx4 Activity Assay

Bone marrow, spleen or MEL cells were lysed in 0.5 mL of lysis buffer (50 mM Tris-HCl, 0.3 mM KCl, 1 mM EDTA, 10% glycerol, 5 mM TCEP, 1% Triton X100, protease inhibitory mix (SERVA, Heidelberg, Germany), pH 7.5). After centrifugation at 10,000× *g* for 10 min the supernatants were used for Gpx4 activity assays. Gpx4 activity was assayed spectrophotometrically employing the coupled optical test [16]. The assay mixture consisted of 1 mL 0.1M Tris/Cl buffer, pH 7.4, containing 5 mM ethylenediaminetetraacetic acid, 0.1% Triton X-100, 0.2 mM NADPH, 3 mM glutathione, and 1 U glutathione reductase. Aliquots of 10,000 g lysate supernatants of MEL cell homogenates (mock-transfected or Gpx4-deficient cells), bone marrow cells or spleen (wild-type or *Gpx4*^+/U46A^ mice) were added, and the assay mixture (absence of substrate) was preincubated at 37 °C for 5 min. Then, the reaction was started by the addition of 25 µM phosphatidylcholine hydroperoxide, and the decrease in absorbance at 340 nm was measured (molar extinction coefficient for NADPH of 6.22 × 10^6^ (M × cm)^−1^).

### 4.11. Ex Vivo Erythropoiesis

#### 4.11.1. Cultivation of Murine Erythroid Progenitors

Mouse erythroblasts were grown from fetal livers as described by von Lindern [38]. Fetal livers of day 12.5 mouse embryos (wild type *Gpx4*^+/+^ or *Gpx4*^+/U46A^) were resuspended in 1 mL serum-free stem cell expansion medium (STEMPRO^TM^-34 SFM Complete Medium; Life Technologies GmbH, Darmstadt, Germany). Cells were passed through a 70 µm Nylon cell strainer (BD Biosciences, Erembodegem, Belgium). The washed cells were seeded into STEMPRO^TM^-34 SFM medium containing 50 ng/mL recombinant murine stem cell factor (SCF, PromoCell GmbH, Heidelberg, Germany), 1 U/mL human erythropoietin-alpha (PromoCell GmbH), and 1 µM dexamethasone (PromoCell GmbH). The proerythroblast culture was expanded for five days keeping 0.7–1 × 10^6^ cells/mL by daily partial medium changes with the addition of fresh factors.

#### 4.11.2. Induction and Analysis of Terminal Differentiation

Terminal differentiation was performed as described by van Lindern [38]. Erythroblasts were washed twice in PBS and seeded at 1.5 × 10^6^ cells/mL in STEMPRO^TM^-34 SFM medium containing 5 U/mL erythropoietin-alpha and 0.5 mg/mL iron-loaded human Transferrin, (Sigma-Aldrich). Differentiating erythroblasts were maintained between 2–4 × 10^6^ cells/mL. Erythroblasts at various stages of differentiation were counted, cytocentrifuged onto glass slides, and stained with 3,3′-dimethoxybenzidine (Sigma) for hemoglobin [46]. The cell morphology was analyzed by staining with histological dyes (Giemsa stain, Roth).

### 4.12. Blood Analysis and Organ Collection

For diagnostic blood analysis and organ preparation mice were sacrificed by cervical dislocation after isoflurane anesthesia. Blood was collected by cardiac puncture into EDTA-containing tubes. The quantification of the basic blood parameters was carried out at the Institute for Veterinarian Diagnostics (Berlin, Germany) using a hematology analyzer (XT-2000iV, Sysmex, Landskrona, Sweden). To isolate bone marrow cells, the femur bones were prepared. Both the ends of the bones were cut off using a scalpel, and the bone marrow cells were eluted from the bone marrow cavity with 10 mL phosphate-buffered saline (PBS). Cells were washed once with 1 mL of PBS and pelleted by centrifugation (800× *g*).

### 4.13. Statistical Analysis

Statistical analyses were carried out with SPSS 23 (IBM, Armonk, NY, USA) software package and the results are presented as medians. For experiments, in which the data are not normally distributed two-tailed Mann/Whitney U test was employed. Significance was accepted at *p* < 0.05. Within the boxplots the black vertical lines indicated the median. The 25th and the 75th percentile are visualized as upper and lower box limits. For normally distributed data the Students *t*-test was used.

## 5. Conclusions

RNAi-induced expression silencing of Gpx4 in mouse erythroleukemia cells delays in vitro erythropoiesis and these data indicate that Gpx4 plays a role in this process. On the other hand, mice expressing heterozygously a catalytically inactive mutant of Gpx4 (*Gpx4*^+/Sec46Ala^ knock-in mice) do not show a defective erythropoietic phenotype and similar results were obtained in ex vivo erythropoiesis studies using embryonic erythroid precursors. Thus, heterozygous expression of a catalytically inactive Gpx4 mutant does not compromise in vivo erythropoiesis.

## Figures and Tables

**Table 1:** Primer sets used for quantitative real-time PCR.

Amplicon	Forward/Reverse	Gene ID
Gapdh	5′-CCATCACCATCTTCCAGGAGCGA-3′ 5′-GGATGACCTTGCCCACAGCCTTG-3′	14433
mGpx4	5′-GAGATGAGCTGGGGCCGTCTGA-3′ 5′-ACGCAGCCGTTCTTATCAATGAGAA-3′	625249
m + cGpx4	5′-CGCCTGGTCTGGCAGGCACCA-3′ 5′-ACGCAGCCGTTCTTATCAATGAGAA-3′	625249
nGpx4	5′-AGTTCCTGGGCTTGTGTGCATCC-3′ 5′-ACGCAGCCGTTCTTATCAATGAGAA-3′	625249
Epo	5′-CACCCTGCTGCTTTTACTCTCCTT-3′ 5′-CTTCTGCACAACCCATCGTGACAT-3′	13856
Hba-a1	5′-ATGGTGCTCTCTGGGGAAGACAAA-3′ 5′-TCATCGAGGTGGCCTGCAGCATT-3′	15122
Alas2	5′-ATGGTGGCAGCAGCTATGTTGCTA-3′ 5′-CTTGAACTTCTGGAGCTGCCCTC-3′	11656

## Data Availability

Not applicable.
